# Trends of ambulatory oral anticoagulant prescription in five major cities of China, 2012–2017

**DOI:** 10.1186/s12913-020-5072-3

**Published:** 2020-03-12

**Authors:** Zhenwei Yu, Lingyan Yu, Chunlei Shan

**Affiliations:** 1 Sir Run Run Shaw Hospital, College of Medicine, Zhejiang University, 3rd East Qingchun Road, Hangzhou, 310016 China; 2 The Second Affiliated Hospital, Zhejiang University School of Medicine, Hangzhou, China

**Keywords:** Warfarin, Rivaroxaban, Dabigatran, Atrial fibrillation, Orthopedist

## Abstract

**Background:**

The introduction of non-VKA oral anticoagulants (NOACs) has changed the landscape of preventing thromboembolism events in many countries. However, the prescription trends of oral anticoagulant (OAC) in China are still unclear, which were evaluated in this study through data extracted and summarized from 5 major cities as representatives.

**Methods:**

This study was designed as a time-series study which was based on pharmacy prescription data. Analysis was performed on yearly aggregated visits and expenditure. The results were also stratified by indications and specialties.

**Results:**

A total of 189,006 prescriptions of 67 hospitals in 6 years were included in the study. The average growth rates of overall visit and expenditure of OAC were 15.8 and 57.5%, respectively. The share of warfarin decreased and NOACs had taken 92% of cost, covering 28% of patients in 2017. The more frequently used NOACs were rivaroxaban and dabigatran. The use of OAC was differed by indication and specialty.

**Conclusion:**

The use of NOACs was found increasing rapidly in both visits and cost, sharing a majority of cost with a minority of patients. Attentions should be paid on the rational use of NOACs.

## Background

Thromboembolism events associated with atrial fibrillation (AF) and venous thromboembolism are leading causes of morbidity and mortality all over the world [1, 2]. Warfarin, the primary Vitamin K antagonist (VKA) in the market, has been the unique oral anticoagulant (OAC) for stroke prevention for decades. However, the introduction of non-VKA oral anticoagulants (NOACs) has changed the landscape of preventing thromboembolism events in many countries [3–7]. In China, three NOACs (rivaroxaban, dabigatran and apixaban) were approved sequentially for clinical use since 2009. Considering the high price of NOACs in China, the cost-utility of NOACs should be carefully re-evaluated [8]. It can be foreseen that there is a great demand of NOACs in China due to the high burden of diseases needing anticoagulation therapy [9, 10]. However, the prescription trends of oral anticoagulant in China are still unclear, which were evaluated in this study through data extracted and summarized from 5 major cities as representatives.

## Methods

### Study design

This study was designed as a time-series study which was based on pharmacy prescription data and was run under the guide of the statement of Reporting of Studies Conducted using Routinely-collected Data (RECORD statement) [11].

### Data collection

The prescriptions were extracted from database of Hospital Prescription Analysis Corporation Program of China [12]. The database contained participated hospitals’ prescription information of 40 random days per year. All outpatient OAC prescriptions were included from hospitals locating in 5 major regions of China (Beijing, Shanghai, Hangzhou, Guangzhou and Chengdu). These 5 cities locate in the north, east, south and west of China respectively, covering a wide area and having a total population of more than 800 million, thus the data were nationally representative. The prescriptions from hospitals which did not participate the program continuously between 2012 and 2017 were excluded. The prescription data included unique prescription code and patients’ code, sex, age, date, location and hospital code, diagnosis, drug generic name and price of drugs. Data with missing field was not included for the calculation involving the missed field.

Sample size was not estimated before data extraction. All prescriptions met the inclusion criteria were included since excessive data could be handled by software easily. It should be noted that this study included prescriptions on specific days and didn’t follow up the same patient group over the study period.

### Primary measurements and data processing

Our primary analysis units were treatment visit and expenditure, and the definition of treatment visit is an ambulatory OAC prescription prescribed, no matter the prescription is the first or renewal. The total expenditure was calculated by add up all the prices of OACs together. The cost per visit was obtained by dividing total expenditure by visits. The study was limited to patients receiving warfarin and 3 NOACs (rivaroxaban, dabigatran and apixaban), aged more than 18. Analysis was performed on yearly aggregated visits and expenditure. The results were also be analyzed by indications and specialties. It should be noted that when assessing OAC use by indication, the retrieved prescriptions cannot clearly distinguish valvular and non-valvular AF.

### Statistical analysis

Trends in proportions will be tested by log-linear analysis. Other trends will be analyzed by Mann-Kendall trend test. All the statistical analysis will be run in R(3.5.0).

## Results

### Characteristics of prescriptions

**Fig. 1:**
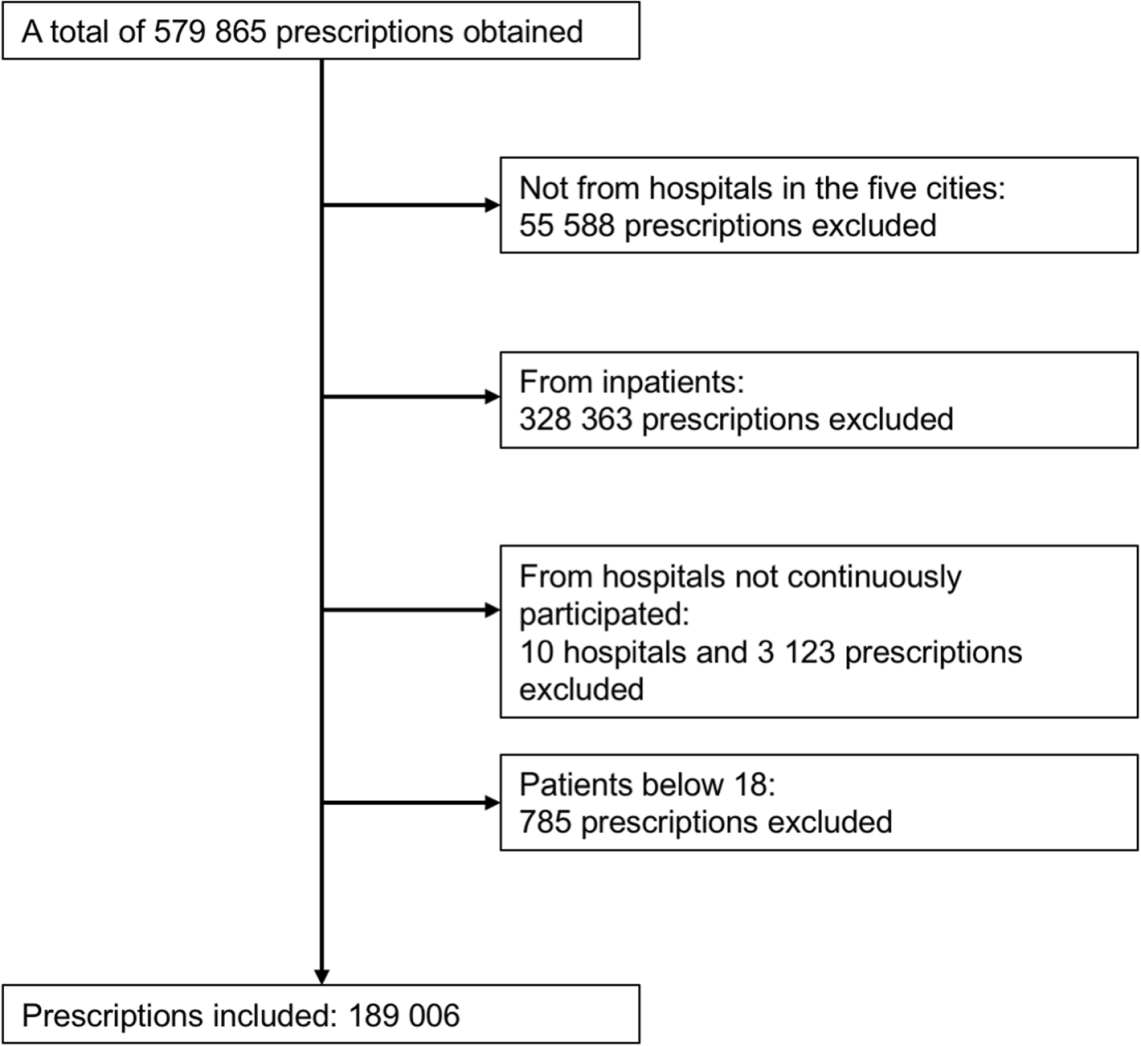
Flowchart of data cleaning

The data cleaning process was shown in Fig. 1. As shown in Table 1, a total of 189,006 prescriptions of 67 hospitals in 6 years were included in the study. In all the included prescriptions, 48.7% were of male. The age ranged from 18 to 117, with an average age of 56.

**Table 1:** Total outpatient visits prescribed with oral anticoagulant of each city during 6 years

	hospital	visits					
		2012	2013	2014	2015	2016	2017
Beijing	17	5657	6214	7026	7802	8789	9443
Shanghai	16	4185	5075	5540	6739	8149	11,989
Hangzhou	12	3816	4396	4918	5262	6078	6776
Guangzhou	12	4258	5455	6118	6368	7691	9389
Chengdu	10	3519	4096	4963	5789	6450	7056

### Overall trends of oral anticoagulant use and expenditure

The overall OAC use increased from 21,435 visits in 2012 to 44,653 visits in 2017, driven by an average growth rate of 15.8% per year (*P* < 0.05). Among all the visits, the percent of patients receiving warfarin declined from about 98% in 2012 to about 72% in 2017 (*P* < 0.05).

**Fig. 2:**
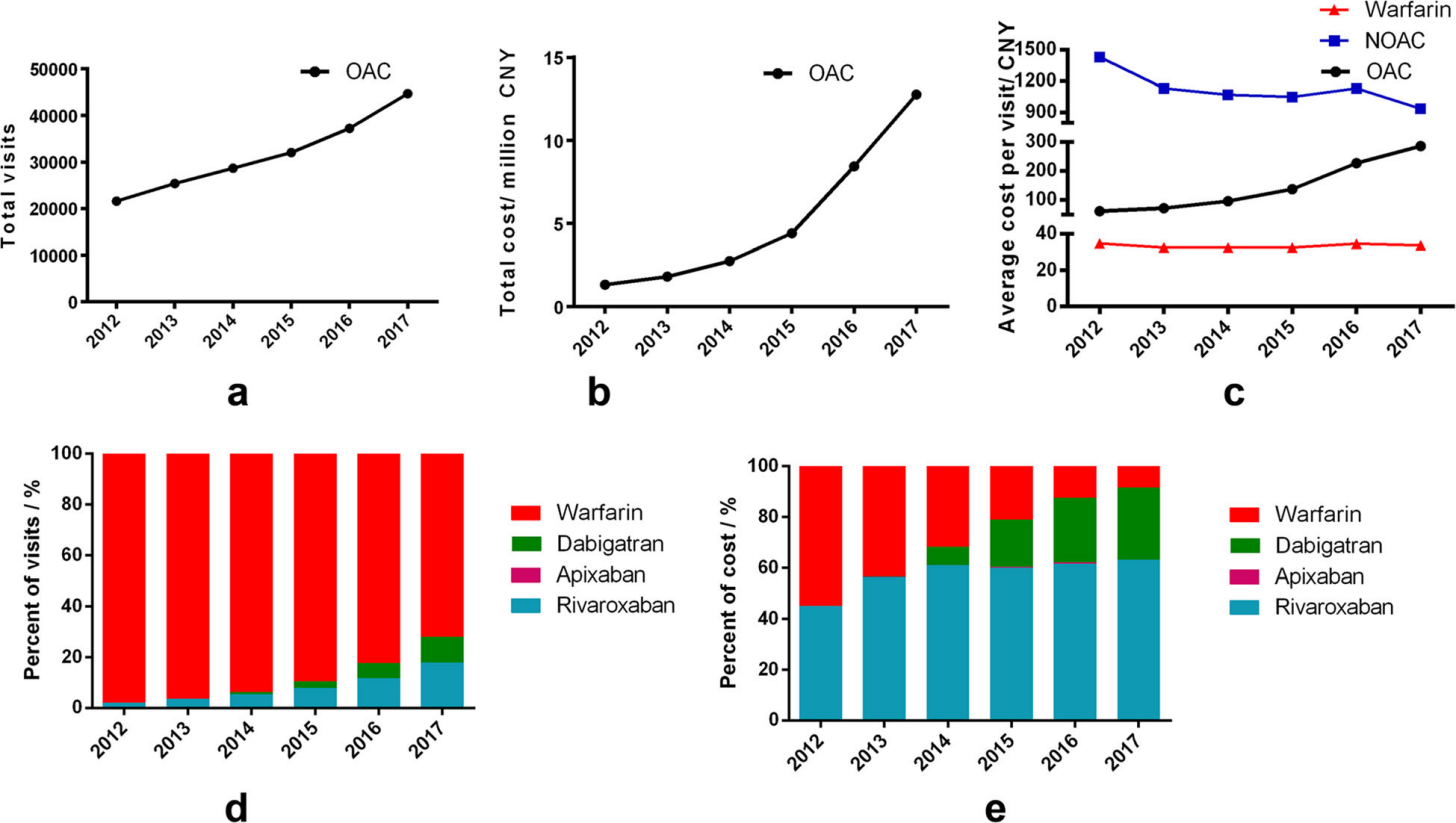
The overall trends of ambulatory anticoagulant use and expenditure in 67 hospitals from 5 cities of China. **a**: Trends of yearly total visits; **b**: Trends of yearly total cost; **c**: Trends of average cost per visit of warfarin, NOACs and OAC; **d**: Trends of percentage of visits taking different OAC; **e**: Trends of percentage of cost taking different OAC

While the total expenditure of OAC seemed to increase more rapidly at an average growth rate of 57.5%. The average cost per visit of OAC kept on increasing during the study period (*P* < 0.05), but the average cost of warfarin remained constant (*P* > 0.05) and of NOACs even decreased (*P* < 0.05). However, the average cost of NOACs at 2017 was about 30 times than the cost of warfarin (Fig. 2). While the percent of cost of warfarin declined with a bigger extent. Warfarin had taken a share of cost at 8% in 2017 and NOACs had taken 92% of cost, covering 28% of patients. The more frequently used NOACs were rivaroxaban and dabigatran, while apixaban took a minimal share of NOACs.

### OAC use by indication

**Fig. 3:**
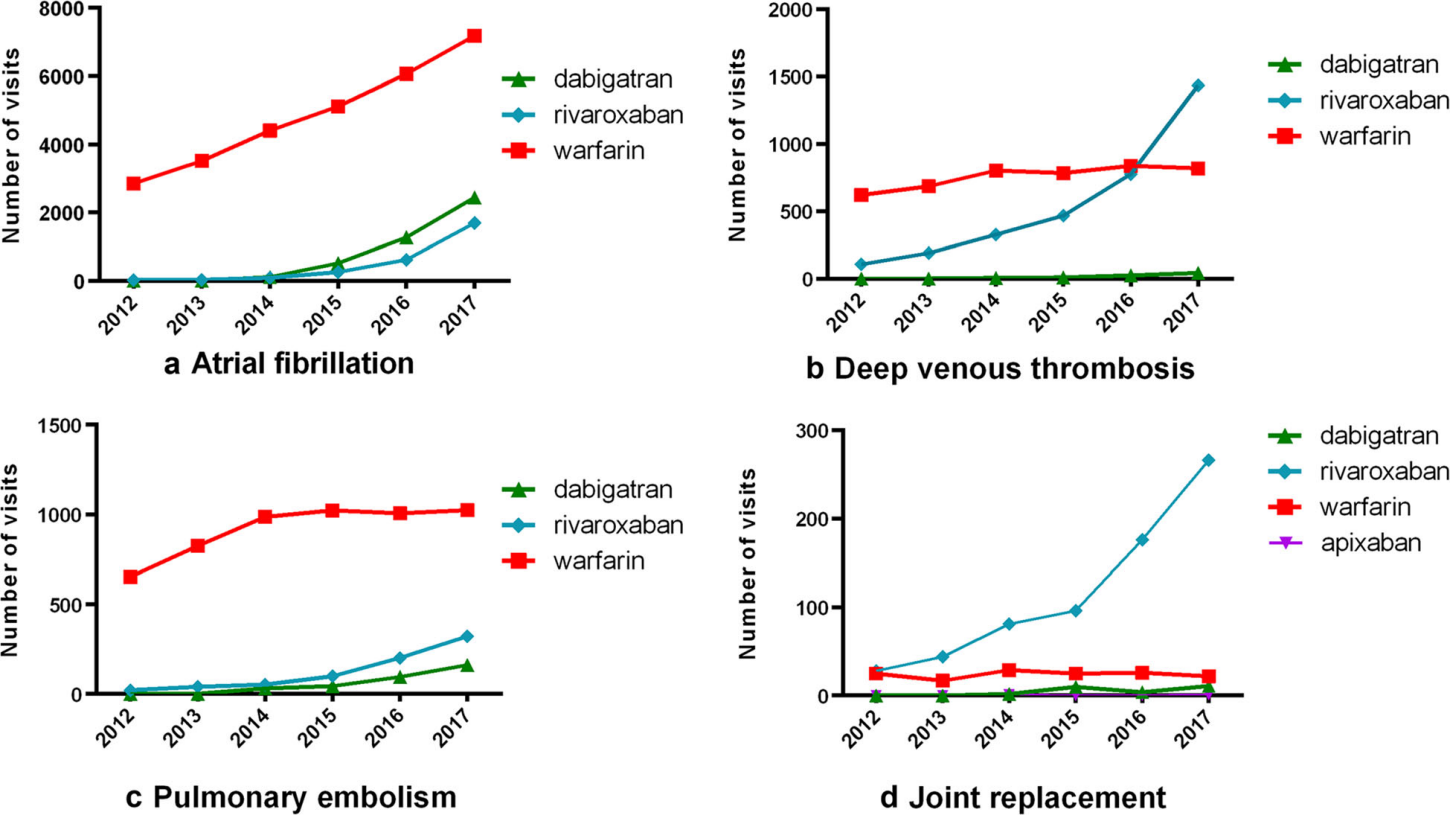
Trends of visits of each oral anticoagulant in 4 common indications

Four diseases, AF, pneumonia embolism (PE), deep vein thromboembolism (DVT) and joint replacement, which need anticoagulation therapy were selected and analyzed for OAC use (Fig. 3). For AF and PE, the most prescribed OAC was warfarin. The visits of AF patients receiving warfarin kept increasing, while which of PE remained constant in the recent years. For patients with DVT, the use of rivaroxaban increased rapidly, and the visits of rivaroxaban user exceed warfarin in 2017. For joint replacement patients, the use of rivaroxaban was close to warfarin at the beginning of study duration and then became the major used OAC at the end of the study gradually. Rivaroxaban was the most frequently prescribed NOAC except in AF patients. Apixaban was rarely prescribed.

### OAC use by specialty

**Fig. 4:**
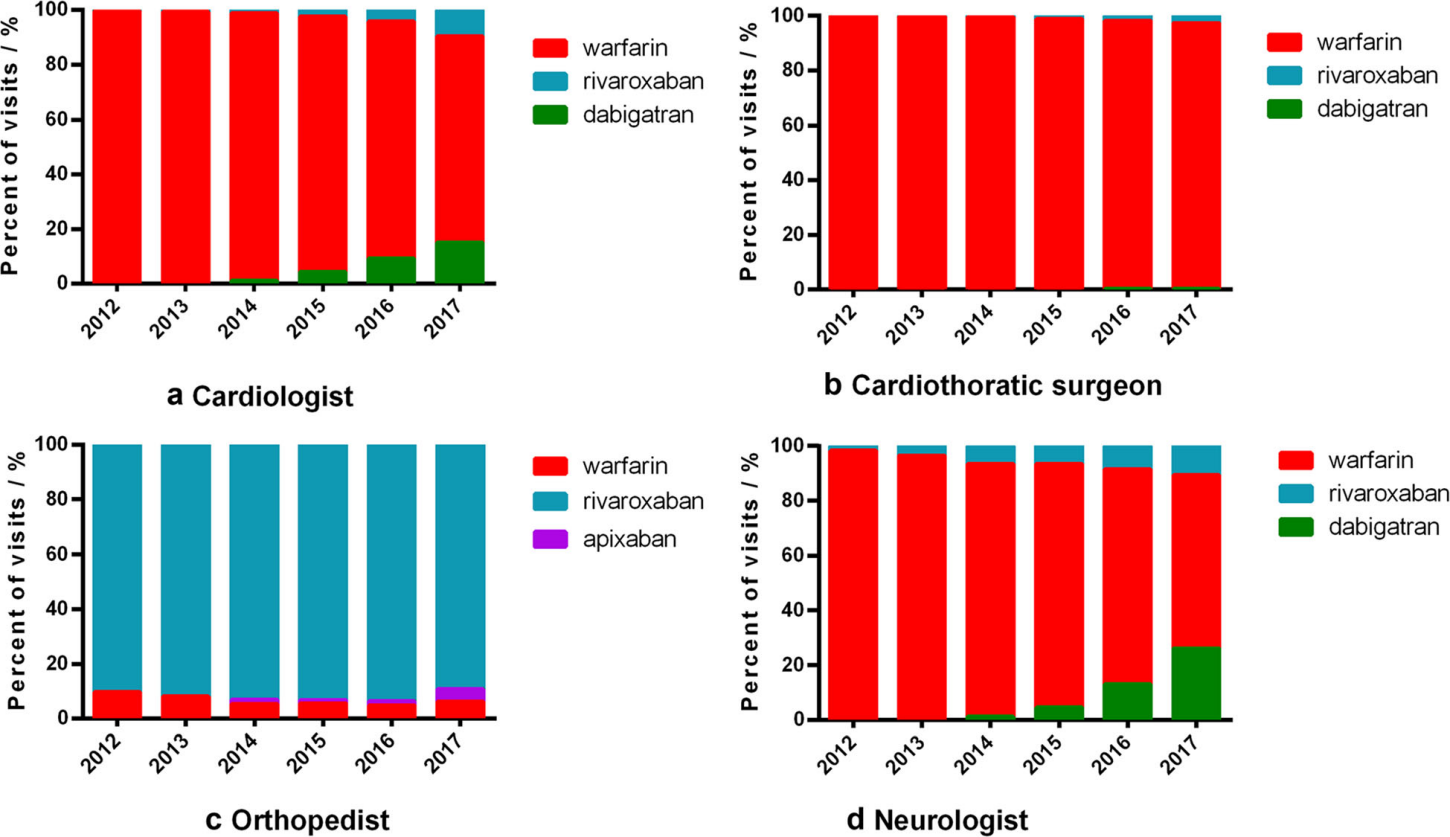
Percent trends of visits of each oral anticoagulant in 4 specialists

The use of OAC was stratified by specialties (Fig. 4). The orthopedists seemed to embrace NOACs in the first place by whom rivaroxaban was the most frequently prescribed, and the use of apixaban increased but also took a minor portion. Cardiothoracic surgeons remained using warfarin as a major option during the 6 years and the portion of NOACs was small. Warfarin used by cardiologists and neurologists decreased but still took the major proportion. The use of rivaroxaban and dabigatran were increasing rapidly in the recent years, and dabigatran was more than rivaroxaban.

## Discussion

The trends of OAC used by patients in 5 major cities of China were investigated through pharmacy prescription data in this study. It could be found that the number of patients receiving OAC and the total cost of OAC both increased rapidly. The percentage of patients who receiving warfarin kept on decreasing, but warfarin was still the most frequently prescribed OAC. Although NOACs took a minor portion in patient number, it became the major component of OAC cost.

The trends of OAC use in China, decreased warfarin and increased NOACs, was similar to other countries. According to the experience of these countries, the use of NOACs will still increase in next several years. For patients who are not able to monitor international normalized ratio (INR) frequently or reach a stable INR, it would be beneficial to switch warfarin to NOACs [13]. However, switching from warfarin to NOACs may bring no benefit to patients who had stable anticoagulation [14]. Holding a small number of patients, NOACs have revealed a higher average cost per patient and occupied a larger part of total cost in OAC nowadays. The average cost of NOAC declined during the study period, that may due to the increased use of a cheaper NOAC, dabigatran. However, the prices of NOAC were much higher than that of warfarin. The increasing use of NOACs had elevated the average cost of OAC. It will exert more pressure on both patients and health insurance system, therefore NOACs should be used after balancing the benefit, risk and cost carefully [15].

As the first NOAC applied to AF patients, rivaroxaban was the most popular at one time, however, rivaroxaban’s market share had been surpassed by dabigatran in recent years. This may due to the accumulated clinical evidences, which showing the priority of dabigatran over rivaroxaban in AF patients [16–18]. By contrast, rivaroxaban strengthened the market in joint replacement patients. It was the first NOAC approved for thromboprophylaxis in patients undergoing joint replacement surgery and was recommended by guidelines [19]. The use of apixaban was rare in China. Orthopedists had rich experience in rivaroxaban and would not switch to apixaban without clinical evidence of superiority. Higher price and more frequent dosing were both apixaban’s shortages [20]. More unfortunately, apixaban has not been approved for AF by Chinese government yet.

The choice of OAC differed among specialists. Orthopedists were the first and most widely embracing group of NOACs which may due to the early approval for orthopedic indication and less NOACs contraindication in these patients. NOACs were only suitable for non-valvular AF patients, thus the cardiothoracic surgeons had the lowest rate of NOAC use.

There are still some limitations of this study. The prescription data didn’t provide the outcomes of anticoagulation therapy. Patients who need but have not received OAC can not be included. The source data were from hospitals which participated in the program and sampling bias may exist. The cost for warfarin only includes the drug cost. Additional cost for taking warfarin, such as monitoring of INR, was not included.

## Conclusion

The use of NOACs was found increasing rapidly in both visits and cost, sharing a majority of cost with a minority of patients. Patients and health insurance system have to face the rapid growth of anticoagulation expenditure. Cost-utility study of NOACs is needed for drug provider, consumer and policy maker. Attentions should be paid on the rational use of NOACs for no benefit of switching to NOACs in warfarin stable anticoagulation patients.

## Data Availability

The datasets analyzed during the current study are available from the corresponding author upon reasonable request.

## Abbreviations

AF: Atrial fibrillation
DVT: Deep vein thromboembolism
NOAC: Non-Vitamin K oral anticoagulant
OAC: Oral anticoagulant
PE: Pneumonia embolism
